# Relationship between cytochrome P450 polymorphisms and prescribed medication in elderly haemodialysis patients

**DOI:** 10.1186/s40064-016-1986-y

**Published:** 2016-03-22

**Authors:** Krystina Parker, Willy Aasebø, Tore Haslemo, Knut Stavem

**Affiliations:** Medical Division, Department of Nephrology, Akershus University Hospital, 1478 Lørenskog, Norway; Institute of Clinical Medicine, University of Oslo, Oslo, Norway; Department of Psychopharmacology, Diakonhjemmet Hospital, Oslo, Norway; Medical Division, Department of Pulmonary Medicine, Akershus University Hospital, Lørenskog, Norway; HØKH, Department of Health Services Research, Akershus University Hospital, Lørenskog, Norway

**Keywords:** Cytochrome P450, Elderly, Haemodialysis, Medication

## Abstract

**Background:**

Elderly patients on haemodialysis have a high prevalence of polypharmacy and are at risk of drug-related complications. More than 80 % of all prescribed drugs are metabolized by the cytochrome P450 (CYP) enzyme system. The aims of this study were to describe the prevalence of polymorphism in three CYP isoenzymes and the relationship between CYP polymorphism and prescribed drugs.

**Methods:**

Fifty-one elderly haemodialysis patients aged ≥65 years were included. CYP-genotyping was carried out in whole blood by a real-time PCR method for detecting common variant alleles in *CYP2C9*, *CYP2C19* and *CYP2D6*. The allele frequencies were calculated using the Hardy–Weinberg equation.

**Results:**

The overall prevalence of CYP polymorphisms (heterozygous and homozygous) was 77 %. The prevalence of heterozygous carriers of variant alleles coding for defective *CYP2D6*, *CYP2C9* and *CYP2C19* was 64, 22 and 55 %, respectively; the prevalence of homozygous carriers was 6 % for each of the *CYP2D6*, *CYP2C9* and *CYP2C19* enzymes. The prevalence of the CYP2D6*6, CYP2D6*9 and CYP2D6*41 variant alleles did not differ (*p* = 0.31) from that in a European Caucasian reference population. Twenty-three patients (45 %) had at least one CYP mutation and used drugs that are metabolized by the CYP isoenzymes. Metoprolol and proton-pump inhibitors were the most commonly used drugs that could be affected by a heterozygous or homozygous mutation.

**Conclusions:**

Polymorphisms of *CYP2C9*, *CYP2C19* and *CYP2D6* are common in elderly haemodialysis patients. Many of these patients have a phenotype with altered CYP enzyme activity and could benefit from close drug monitoring or a drug switch.

## Background

Patients with end-stage renal disease (ESRD) are treated with various medications for their kidney disease and associated comorbidities. The interindividual variability in drug response represents a clinical challenge. Factors such as age, smoking, fluid balance and other diseases influence drug effects (Samer et al. 2013). In addition, genetic variations in the cytochrome P450 (CYP) enzyme system contribute to variability in drug response through altered metabolism. More than 80 % of all medications in use today are metabolized by the CYP enzyme system, which is a microsomal superfamily involved in the biosynthesis and degradation of endogenous compounds, chemicals, toxins and drugs (Trescot 2013). The most important enzymes for drug metabolism are *CYP2C9*, *CYP2C19*, *CYP2D6* and *CYP3A4*.

The *CYP2C9* enzyme partly determines warfarin metabolism and activity, and patients with alleles CYP2C9*2 and CYP2C9*3 require lower doses to avoid bleeding (Aithal et al. 1999; Samer et al. 2013; Beyth et al. 2000; Higashi et al. 2002; Sanderson et al. 2005). Therefore, a genotype-guided dosing of warfarin has been suggested (Pirmohamed et al. 2013). The *CYP2C19* enzyme metabolizes common drugs such as clopidogrel, proton-pump inhibitors and antidepressants. The CYP2C19*2 and CYP2C19*3 alleles are associated with the clinical efficacy of clopidogrel (Umemura et al. 2008; Brandt et al. 2007), and hence also with the risk of cardiovascular events (Mega et al. 2010). The *CYP2D6* enzyme is highly polymorphic and metabolizes various psychotropic agents such as antidepressants, neuroleptics and opioids. The *CYP2D6* poor metabolizer (PM) phenotype is associated with more frequent adverse drug reactions (Chen et al. 1996; Chou et al. 2000; Kirchheiner et al. 2004a, b) and varying responses to analgesics such as codeine, tramadol and oxycodone (Samer et al. 2010a, b; Brousseau et al. 2007).

The prevalence of mutations in these three CYP isoenzymes has not previously been reported for patients with ESRD. However, some studies have documented that chronic renal failure decreases drug metabolism by lowering the activity in the CYP enzyme system by circulating uremic toxins, increased parathyroid hormone and markers of inflammation (Dreisbach and Lertora 2008; Guevin et al. 2002; Michaud et al. 2005, 2006; Renton 2004).

The aims of this study involving elderly haemodialysis patients were to (1) describe the prevalence of CYP polymorphisms, (2) measure the allele frequency of each CYP isoenzyme and (3) determine the prescribed medication for patients with altered enzyme activity through CYP polymorphisms.

## Results

The characteristics of the 51 included patients and the most commonly used medications are given in Table 1. In all 47 (92 %) patients used medications metabolised by the CYP enzyme system; of whom 23 (45 %) had genetically altered metabolism via at least one of the tested CYP isoenzyme.

**Table 1:** Characteristics of the study population (*n* = 51)

Gender, male	39 (77)
Age, years, median (range)	74 (65‒89)
Charlson comorbidity index, median (range)	5 (2–9)
Time in haemodialysis, months, median (range)	8 (0–108)
Urea clearance, Kt/V	1.47 ± 0.29
Arteriovenous fistula	29 (57)
Haemodialysis catheter	22 (43)
*Diagnosis*	
Glomerulonephritis	3 (6)
Nephrosclerosis	18 (35)
Diabetic nephropathy	7 (14)
Post-renal disease^a^	8 (16)
Other^b^	8 (16)
Unknown	7 (14)
*Medications*	
Diuretic drugs	38 (75)
Statins	31 (61)
Beta-blockers	29 (57)
Proton-pump inhibitors	19 (37)
Analgesics	17 (34)
ACE inhibitor^c^/angiotensin II receptor antagonist	14 (28)
Warfarin	11 (22)
Benzodiazepines	7 (14)
Tricyclic antidepressants	3 (6)
Number of medications, median (25th–75th percentile)	13 (12–14)

Except where stated otherwise, the data are number (%)

^a^Post-renal disease = hydronephrosis, kidney stone disease, retroperitoneal fibrosis

^b^Other = kidney cancer, loss of kidney graft, amyloidosis, poisioning, antiglomerular basement membrane nephritis

^c^ACE inhibitor = angiotensin-converting enzyme inhibitor

In total, 64 and 6 % of the patients were heterozygous and homozygous carriers of variant alleles encoding defective *CYP2D6*, respectively; these corresponding proportions for the alleles encoding defective *CYP2C9* were 22 and 6 %, and for the alleles encoding defective *CYP2C19* they were 55 and 6 %. Of the 51 patients, 39 (77 %) had one or more CYP enzyme defects, defined as either heterozygous or homozygous. Furthermore, 16 patients (31 %) had two CYP defects (Table 2).

**Table 2:** Polymorphisms of the CYP enzymes *CYP2C9, CYP2C19* and *CYP2D6* (*n* = 51)

Enzyme	Enzyme activity	Genotype	Number (%)	95 % CI, %
*CYP2C9*	Normal	*1/*1	37 (73)	58–84
	Intermediate	*1/*2, *1/*3	10 (20)	11–35
	Poor	*2/*2, *2/*3,*3/*3	4 (8)	1–16
*CYP2C19*	Normal	*1/*1	20 (39)	26–54
	Approximately normal	*1/*17, *2/*17	18 (35)	22–50
	Intermediate	*1/*2,*1/*3	10 (20)	9–33
	Poor	*2/*2	2 (4)	0–13
	Increased	*17/*17	1 (2)	0–10
*CYP2D6*	Normal	*1/*1	13 (26)	14–40
	Approximately normal	*1/*9, *1/*41	10 (20)	10–33
	Intermediate	*1/*3, *1/*4, *1/*5, *1/*6, *9/*9, *10/*10, *10/*41, *4/*41	24 (47)	33–62
	Poor	*3/*4, *4/*4, *4/*5, *5/*6	4 (8)	2–19

95 % CI = 95 % confidence interval

The frequencies of the CYP2C9*1, CYP2C9*2, CYP2C9*3, CYP2C19*1, CYP2C19*2, CYP2C19*3, CYP2C19*17, CYP2D6*1, CYP2D6*3, CYP2D6*4, CYP2D6*5, CYP2D6*6, CYP2D6*9, CYP2D6*10 and CYP2D6*41 alleles in the haemodialysis population are presented in Table 3. The *CYP2D6* enzyme was highly polymorphic. The most frequent inactive allele was CYP2D6*4, followed by CYP2D6*5, CYP2D6*3 and CYP2D6*6. Although the CYP2D6*6, CYP2D6*9 and CYP2D6*41 alleles appeared to be more common in the present study population than among European Caucasians, the difference was not statistically significant (*p* = 0.31).

**Table 3:** Frequencies of *CYP2C9*, *CYP2C19* and *CYP2D6* variant alleles in the haemodialysis patients, and population reference values

CYP enzyme	Allele and activity	Allele	Allele frequency (%)		
			Haemodialysis population (*n* = 51)		Reference populations: Caucasians^a^
			*n*	95 % CI	
*CYP2C9*	Normal	1^a^	82	74–91	78–86
	Decreased	2^a^	8	2–14	8–15
		3^a^	10	3–16	1–8
*CYP2C19*	Normal	1^a^	59	48–69	87
	Decreased	2^a^	21	13–28	13
		3^a^	0	0–3	0
	Increased	17^a^	20	12–27	20
*CYP2D6*	Normal	1^a^	54	45–63	33–84
	None	3^a^	2	0–5	0–3
		4^a^	21	13–28	11–29
		5^a^	4	0–8	1–7
		6^a^	2	0–5	1–2
	Decreased	9^a^	4	0–9	0–3
		10^a^	3	0–7	1–6
		41^a^	11	5–16	8–10^b^

^a^Data from McGraw et al. (McGraw and Waller 2012)

^b^Data from Preissner et al. (Sachse et al. 1997)

Most patients (47 %) were identified as IM, followed by PM (8 %). Nine single drugs and drugs belonging to two other drug classes were being used by IM or PM patients for all three CYP isoenzymes. Metoprolol was the most prevalent drug among the PMs and IMs of *CYP2D6*, while proton-pump inhibitors were the most frequently used among those for *CYP2C19* (Table 4).

**Table 4:** Activity of CYP enzymes and prescribed drugs potentially influenced by CYP polymorphisms (*n* = 51)

CYP enzyme	Enzyme activity	Drug	Number
*CYP2C9*	Intermediate (*n* = 11)	Warfarin	1
		Losartan	1
	Poor (*n* = 3)	Warfarin	1
*CYP2C19*	Intermediate (*n* = 10)	Proton-pump inhibitor	4
		Diazepam	1
	Poor (*n* = 2)	Proton-pump inhibitor	1
		Diazepam	1
	Increased (*n* = 1)	Escitalopram	1
*CYP2D6*	Intermediate (*n* = 24)	Metoprolol	14
		Codeine	3
		Tricyclic antidepressants	2
		Tolterodine	1
		Tramadol	1
	Poor (*n* = 4)	Metoprolol	2
		Bisoprolol	1
		Tricyclic antidepressants	1

## Discussion

To the best of our knowledge, this study is the first to identify the prevalence of three CYP isoenzymes (*CYP*2C9, *CYP2C19* and *CYP2D6*) and their allele frequencies in an elderly haemodialysis population, and the first to determine the relationship between homozygous and heterozygous mutations of all CYP isoenzymes and prescribed medications.

The main finding of this study was the high prevalence (77 %) of genetic polymorphisms of three CYP isoenzymes. This finding is in line with a previous report of a high prevalence of the same three CYP isoenzymes in a high-opioid-use population (Tennant 2012) (although this was in a completely different population). However, to the best of our knowledge there is a dearth of prevalence studies for CYP polymorphisms in most common chronic diseases. This is the first study of haemodialysis patients using this approach.

The frequencies of the *CYP2C9*, *CYP2C19* and *CYP2D6* alleles have been reported elsewhere for various populations (Waade et al. 2014; Molden et al. 2002; Swen et al. 2012; Mega et al. 2011; Tamura et al. 2011). CYP2D6*3, CYP2D6*4, CYP2D6*5 and CYP2D6*6 are known to be inactive alleles, and the CYP2D6*4 allele is the most common (Bradford 2002; Gaedigk et al. 1999). These four inactive alleles accounted for 29 % of the *CYP2D6* alleles in the present study, which is comparable with a percentage of 26 % reported for a European general population (Bradford 2002). The proportion of PMs in the elderly haemodialysis population in the present study is comparable with those reported previously (Bradford 2002; McGraw and Waller 2012). However, the CYP2D6*6 and CYP2D6*9 alleles were more prevalent in the present population than in Caucasian and in Central and South American Indian populations (Bradford 2002; Jorge et al. 1999; Marez et al. 1997; Griese et al. 1998; Gaedigk et al. 1999; Sachse et al. 1997). The previously reported frequencies of the CYP2C19*2, CYP2C19*3 and CYP2C19*17 alleles are similar to those reported here (Rudberg et al. 2008; Sim et al. 2006; Kurzawski et al. 2006).

A study involving non-institutionalized patients aged >60 years found that medications metabolized by CYP systems were used by 62 % of patients (Cabrera et al. 2009), versus 92 % in the present study. Elderly patients are known to be exposed to higher drug concentrations for a given dose compared to younger adults (Waade et al. 2012), due to multiple factors such as reductions in hepatic blood flow, cardiac output and renal function. Both renal failure and genetically reduced or absent enzyme activity increase the vulnerability to side effects. A recent report confirmed the effects of age on exposure to antidepressants in patients with the *CYP2C19* and *CYP2D6* PM genotypes (Waade et al. 2014).

This study was subject to some limitations. First, there was no comparator healthy control group of elderly subjects, which would have enabled a better understanding of the genetic variation of the three CYP isoenzymes and their allele frequencies. Second, as no measurements of drug levels were performed to investigate the effect of altered CYP isoenzymes on the elimination of the drugs, the study was unable to show the practical impact of these polymorphisms in this population. Finally, most of the study patients were European Caucasians, hence limiting the generalizability of the findings.

In general, the use of pharmacogenetic testing in clinical settings has the potential to improve patient outcomes and the long-term cost of care through reduction of polypharmacy and risk of drug-related problems (Dorfman et al. 2013), although the practical implications of detected CYP enzyme polymorphism are not yet clear. Some studies including CYP genotyping have provided useful information about medication dosages, or have led to changes in treatments (Pirmohamed 2014; Molden et al. 2002; Mega et al. 2011; Pettersen et al. 2011). The US Food and Drug Administration has published a list of nearly 100 drugs with a recommendation for genetic testing (FDA 2014). Similarly, the Dutch Pharmacogenetics Working Group guidelines recommend dose adjustments in 5–10 % of drugs prescribed to patients with altered *CYP*2D6 and *CYP*2C19 metabolism, and increased awareness for an adverse drug response in all these patients (Swen et al. 2012). Thus, the finding of a CYP enzyme abnormality does not necessarily require dose adjustment.

## Conclusions

In conclusion, a high prevalence of three CYP isoenzymes was found in elderly haemodialysis patients at the level of the general population. About 45 % of these patients had one or more mutations in their CYP isoenzymes and used medications for which the metabolism may be affected by such mutations. These patients may benefit from close drug monitoring or a drug switch. The actual impact of these mutations on drug serum levels is a topic for future studies.

## Methods

### Study population and data collection

This study was performed between July and December 2012 in the dialysis centre at Akershus University Hospital, Norway. This hospital has a catchment area comprising about 480,000 inhabitants. Of the 102 haemodialysis patients screened at the start of the study, 52 were ≥65 years of age and eligible for inclusion. One patient died before assessment; the remaining 51 patients participated in the study.

The following patient data were collected via review of their medical records: medical history, comorbidities (evaluated using the Charlson comorbidity index), dialysis treatment quality index (quantified in units of Kt/V), haemodialysis access and medication history. Blood samples were drawn at the start of dialysis treatment on the day of a regularly scheduled haemodialysis session.

Our institute ethical committee evaluated the study protocol and approved the study. All participants gave their consent at the start of the study.

### Genotyping of CYP enzymes

Venous blood samples (with EDTA as anticoagulant) were genotyped at the Centre for Psychopharmacology, Diakonhjemmet Hospital, Norway. A real-time PCR method was applied using mutation-specific TaqMan probes (Life Technologies, Foster City, CA, USA) (Schaeffeler et al. 2003). Genomic DNA was extracted instrumentally from leukocytes prior to applying the PCR using a MagNA Pure LC DNA Isolation Kit I (Roche Diagnostics, Oslo, Norway). The genotyping assay included detection of the single-nucleotide polymorphisms specific for the following variant alleles: CYP2C9*2 and CYP2C9*3; CYP2C19*2, CYP2C19*3, CYP2C19*4 and CYP2C19*17; and CYP2D6*3, CYP2D6*4, CYP2D6*6, CYP2D6*9, CYP2D6*10 and CYP2D6*41. In addition, copy-number analyses was implemented to establish the presence of *CYP2D6* gene deletion (CYP2D6*5) or multiplication. The absence of mutated alleles was interpreted as the presence of the functional wild-type allele (CYP2D6*1). The genotyping assay did not discriminate between functional and non-functional *CYP2D6* multiplications.

### Classification of enzyme activity

A genotype-predicted phenotype was assigned to each patient. For *CYP2D6*, intermediate metabolizers (IMs) were defined as patients carrying two reduced-activity alleles (CYP2D6*9, CYP2D6*10 and CYP2D6*41) or carrying one inactive allele (CYP2D6*3, CYP2D6*4, CYP2D6*5 and CYP2D6*6). Poor metabolizers (PMs) were defined as patients carrying two inactive alleles (CYP2D6*3, CYP2D6*4, CYP2D6*5 and CYP2D6*6). For *CYP2C19* and *CYP2C9*, IMs were defined as patients with one inactive allele (CYP2C19*2, CYP2C19*3, CYP2C9*2, and CYP2C9*3), while PMs were defined as patients carrying two inactive alleles.

### Statistical analyses

Descriptive statistics are presented as median (range or 25th–75th percentiles) or number (%) values as appropriate. The allele frequencies were calculated using the Hardy–Weinberg equation and compared to published general population references for European Caucasians (McGraw and Waller 2012). Groups were compared using Fisher’s exact test. All analyses were carried out using SPSS statistical software (version 19, IBM, SPSS, Chicago, IL, USA). The threshold for statistical significance was set at *p* < 0.05 (two-sided tests). The study was approved by the privacy ombudsman of Akershus University Hospital.

## Abbreviations

CYP: cytochrome P 450
DNA: deoxyribonucleic acid
ESRD: end stage renal disease
IM: intermediate metabolizer
PCR: polymerase chain reaction
PM: poor metabolizer
